# Impact of uteroplacental insufficiency on ovarian follicular pool in the rat

**DOI:** 10.1186/s12958-019-0453-3

**Published:** 2019-01-10

**Authors:** Valentina Pampanini, Kirsi Jahnukainen, Lena Sahlin, Daniela Germani, Antonella Puglianiello, Stefano Cianfarani, Olle Söder

**Affiliations:** 1 NORDFERTIL Research Lab Stockholm, Pediatric Endocrinology Unit, Department of Women’s and Children’s Health, Karolinska Institutet and University Hospital, Visionsgatan 4; J9:30, SE-171 64, Solna, Stockholm, Sweden; 2 Department of Systems Medicine, Tor Vergata University, Rome, Italy; 3 Dipartimento Pediatrico Universitario Ospedaliero “Bambino Gesù” Children’s Hospital – Tor Vergata University, Rome, Italy

**Keywords:** Ovary, Follicle reserve, Intrauterine growth restriction, Developmental origins of health and disease

## Abstract

**Background:**

A low oxygen supply to the fetus causes intrauterine growth restriction and can affect gonadal development of the offspring, having a potential impact on fertility. We investigated histology and gene expression in the postnatal rat ovary after fetal hypoxia induced by uterine artery ligation.

**Methods:**

Sprague-Dawley rats underwent uterine artery ligation at day 19 of gestation. Offspring were sacrificed at 5, 20 and 40 days *post-partum*. Follicles were counted and classified in hematoxylin-eosin stained sections. Gene expression of 90 genes was analyzed by TaqMan® Low Density Array.

**Results:**

A significantly lower number of total and primordial follicles was detected in 20 days *post-partum* intrauterine growth restricted animals. Follicle density was not different at 40 days *post-partum*, suggesting that compensatory mechanisms occurred during the pre-pubertal window. Uterine artery ligation modified the expression of 24 genes involved in different cellular functions, among which proliferation, apoptosis and metabolism.

**Conclusion:**

Ovarian follicle pool was affected by fetal hypoxia in early life, but this effect did not persist in puberty. Genes involved in cellular processes were affected at all ages, potentially implying long-term genetic alterations. Further analyses are needed to elucidate later effects of fetal hypoxia on ovarian function and fertility.

**Electronic supplementary material:**

The online version of this article (10.1186/s12958-019-0453-3) contains supplementary material, which is available to authorized users.

## Background

In the early ‘90s David Barker and colleagues published several epidemiological studies correlating birth weight with metabolic risk and rates of death for cardiovascular diseases in adulthood [1–6]. These observations gave origin to the theory that adult diseases’ predisposition originates in fetal life, as a consequence of a suboptimal intrauterine environment leading to poor growth of the fetus [4]. This theory has been later defined ‘Developmental Origins of Health and Disease’ (DOHaD) hypothesis [7] and supported by a wealth of scientific evidence. Adverse in utero conditions have been proven to permanently shape gene expression, thereby affecting the structure and function of different organs [8]. Pathological events during fetal development encompass maternal malnutrition, placental dysfunction and maternal inflammatory status. Placental insufficiency, leading to a compromised blood flow to the fetus, fetal hypoxia and consequent intrauterine growth restriction (IUGR), is the most common cause of IUGR in Western countries. Smallness for gestational age has been used in clinical studies as a surrogate measure of IUGR, although the two entities may not be the same.

Altered sexual maturation has been reported in low birth-weight/small for gestational age (SGA) children [9–11]. Girls born SGA have higher FSH levels during infancy [12, 13], an earlier onset of puberty [14, 15] and a lower age at menarche [15–17] than those with a birth weight appropriate for gestational age (AGA).

A higher prevalence of anovulation and smaller ovaries at ultrasound measurements were shown in post-menarcheal girls born SGA compared to AGA counterparts [18–20]. In rodents and ovine, IUGR induced by maternal undernutrition caused DNA damage in fetal oogonia [21] and affected folliculogenesis [22, 23]. We have previously shown that IUGR induced by uteroplacental insufficiency (UPI) altered the morphology and the gene expression profile in the testes of postnatal rats [24]. In female rats, UPI was shown to delay pubertal development and to reduce the number of follicles in adulthood [25].

Here, we used the same animal model to investigate the effect of IUGR on the gene expression and the follicle pool in ovaries of rats from neonatal to peripubertal ages.

## Material and methods

### Animals

All protocols for the study were approved by the Committee for Animal Research of Tor Vergata University, Rome, Italy. Animal experiments were performed according to the Guide for the Care and Use of Laboratory Animals of the National Institutes of Health (NIH Publication No. 85–23, revised 1996). All procedures complied with Italian regulations for laboratory animal care, according to the guidelines and under supervision of the Animal Technology Station, Interdepartmental Service Center, Tor Vergata University, Rome, Italy.

The animal model was developed as previously described [24]. Briefly, time-dated Sprague-Dawley pregnant rats (Charles River Laboratories Inc., Italy) were delivered to the animal facilities at least 3 days prior to the first surgery. IUGR was induced by bilateral ligation of the uterine arteries on day 19 of gestation, according to the method described from Wigglesworth in 1964 [26]. Control animals (*shams*) were born from mothers that underwent the same surgical procedure, with the exception of uterine artery ligation. Dams delivered spontaneously during the night between day 22 and 23. Animals were selected as significant IUGR if their birth weight (BW) was more than 2 standard deviations (SDS) below the mean BW of the control litter. Newborn pups were assigned to feeding dams with a maximum number of six offspring *per* litter. Pup weight was recorded on day 0 and thereafter once a week until the end of experiments. However, it was not possible to follow individual animals until sacrifice or until the age of 3 weeks when the ear tag was placed. At 21 days *post-partum* (d*pp*), pups were weaned onto standard rat chow diet (Mucedola S.R.L., Milano, Italy), separated from dams and placed in groups of three to five, with the males and females separated. For the purpose of this study only female pups were evaluated. The results of the analyses on male pups have been reported earlier [24]. Animals were sacrificed through cervical dislocation at postnatal days 5, 20 and 40 d*pp*. These age points represent in the rat the neonatal (end of follicular assembly), juvenile (appearance of the first large antral follicles and atresia of medullary follicles) and peripubertal (first ovulation) periods [27]. In order to ensure biological variability of the observed phenotype, each group of IUGR and *shams* at 20 and 40 d*pp* included six animals from at least three different litters. For the 5 d*pp* old animals, starting material included eight IUGR and four *sham* animals, also derived from three different litters. For all of them body and ovary weight were measured. Gene expression analysis was carried in 6 IUGR and 3 *sham* animals. Histological evaluation was performed in 4 IUGR and 4 *sham* animals.

### Tissue processing and immunohistochemistry-anti-Mullerian hormone (AMH)

At sacrifice, ovaries were immediately excised from euthanized animals and processed as previously described [24]. In brief, for histological purpose, one gonad was fixed in 4% paraformaldehyde (PFA; P/N15812–7, Sigma-Aldrich, MO, USA) overnight at 4 °C, serially dehydrated in increasing concentration of aqueous ethanol followed by 100% butyl acetate (P/N 45860, Sigma-Aldrich, MO, USA) and finally embedded in paraffin (Paraplast X-TRA®; P/N P3808, Sigma-Aldrich) at 61 °C overnight. Ovaries were cut serially to a thickness of five μm, using a Biocut sectioning machine (Reichert-Jung, NY, USA), mounted on microscope slides (P/N10143352, Superfrost Plus, Thermo Scientific, MA, USA) and placed at 37 °C overnight.

For immunohistochemical (IHC) analysis, three sections per ovary corresponding to ~ 25, 50 and 75% points of the entire tissue block (i.e. 25th, 50th and 75th sections when the total number was 100) were stained and evaluated [28]. Tissue sections were dewaxed with xylene (P/N 02080, HistoLab, Gothenburg, Sweden) for 10 min and then serially rehydrated with decreasing concentrations of aqueous ethanol. Antigen retrieval was performed using citrate buffer (pH 6.0) at 95 °C for 10 min. Slides were then incubated with 3% H_2_O_2_ in methanol for 10 min at RT for endogenous peroxidase blocking followed by incubation with 2% horse serum in 1× tris-buffered saline (TBS) for 30 min at RT to prevent nonspecific antibody binding. Afterwards, samples were incubated with primary antibody against AMH (P/N MCA2246, Biorad, CA, USA) or unspecific IgGs (for negative control) dissolved in 2% horse serum in TBS overnight at 4 °C. After washing with TBS plus 0.01% Tween20 (P/N P1379, Sigma Aldrich,), slides were incubated with biotinylated secondary antibody (P/N BA1400, Vector laboratories, CA, USA), and then with avidin-biotin-peroxidase complex prepared using Vectastain ABC kit (P/N PK-6100, Vector Laboratories, CA, USA) for 30 min each at 37 °C. After washing again twice, slides were stained with DAB (P/N SK-4105, Vector Laboratories,) at RT, counter-stained with hematoxylin solution modified according to Gill III, rinsed for 5 min with running tap water, dehydrated with gradually increasing concentrations of ethanol, cleared with xylene and finally mounted using Pertex (Cell Path, Hemel Hempstead, UK) and cover slips.

### Histomorphometry and follicle counts

For histological evaluations, ovarian sections were stained with hematoxylin and eosin. In brief, following dewaxing in xylene and serial rehydration in ethanol, samples were washed twice with distilled water, incubated for 3 min with hematoxylin solution modified according to Gill III and then rinsed with tap water for 20 min, followed by 70 and 95% ethanol baths for 5 min each. Slides were finally incubated with eosin for 30 s, dehydrated with 100% ethanol for 5 min and 100% xylene twice for 5 min, and finally mounted with Pertex (Cell Path, Hemel Hempstead, UK) and cover slips. Three sections per ovary, corresponding to ~ 25, 50 and 75% points of the entire tissue block [28], were selected for follicle quantification. For each section, only follicles with a visible nucleus were counted and classified as follows: (1) primordial follicles (PF), with one layer of flattened pre-granulosa cells (GCs); (2) primary, with two or more cuboidal GC up to one complete layer of cuboidal GCs; (3) secondary, with a least two layers of GCs but no antrum; and (4) tertiary, where the antrum was visible [29, 30]. Two blinded observers evaluated the same sections independently and the results were compared showing an inter-observer concordance above 90%.

*Corpora lutea* (CL) were quantified in the same three sections per animal as an indirect measure of the ovulation rate.

The ovary was approximated to a prolate ellipsoid [31] and the ovarian volumes (mm^3^) extrapolated using the ellipsoid formula 4/3 π a b^2^, where ‘a’ is the length of the entire ovary derived by multiplying the total number of sections (*n*) by 0.005 (representing the thickness of each section), and π b^2^ is the Area of the middle section (A*m*) of the ovary. Therefore, ovarian volume (*V*) = 4/3 * A*m* * n * 0.005.

### RNA isolation and cDNA synthesis

Total RNA was extracted from whole frozen ovaries and stored at − 80 °C using an RNeasy Mini Kit (P/N 74104, Qiagen, Veno, Netherlands). In brief, samples were dissolved in Qiagen lysis buffer and then homogenized twice for 30 s in an ULTRA-TURRAX T25 homogenizer (JANKE and KUNKEL, Staufen, Germany). Subsequently, the supernatant was mixed with half its volume of 70% ethanol. The mixture was then transferred to RNeasy spin columns and processed according to the manufacturer’s protocol.

RNA concentrations were quantified using a single-beam U*V*/*v*is spectrophotometer (P/N 6132000032, Eppendorf, Hamburg, Germany). To generate cDNA, one μg of total RNA was reverse-transcribed on a thermocycler (P/N 4359659, Applied Biosystems, MA, USA) using random hexamers in a total reaction volume of 20 μl. The IScript™ cDNA synthesis kit (P/N 170–8891, Bio-Rad, CA, USA) was used as instructed by the manufacturer.

### TaqMan low-density arrays (TLDAs)

TLDA cards (P/N 4342259, ABI, Hilden, Germany) were used for comparative analysis of gene expression of IUGR and control animals, according to the manufacturer’s protocol, as previously described [24]. Briefly, the TLDA cards are based on TaqMan chemistry where gene expression of a panel of 96 genes is analyzed in one run. The TLDA cards were pre-loaded with 96 TaqMan gene expression assays of importance for proliferation, apoptosis, cellular energy, germ cell and somatic cell function and differentiation, and six endogenous controls assigned for normalization. Gene expression was normalized to the mean of five out of six endogenous controls (Actin beta, Beta-2-microglobulin, Catenin beta 1, Eukaryotic translation elongation factor 1 alpha 1 and Glyceraldehyde-3-phosphate dehydrogenase) of the same sample (dCt), selected according to their stability. Data from one randomly chosen animal were used as a calibrator (ddCT) and data from all other animals were normalized to it. Gene expression was finally presented as relative expression [using the fold change (2 − ddCT) method for calculation].

TaqMan Gene expression Master Mix (P/N 4369510, Applied Biosystems, MA, USA) was used when running the TLDA assays.

### Serum AMH measurement

AMH serum levels were measured in 5, 20 and 40 d*pp* animals by Gen II ELISA Reagent kit REF A79765 and Calibrator kit A79766 (Beckman Coulter) in accordance with manufacturer’s protocol. Detection limit was 0.08 ng/mL and the intra and inter-assay variations were 4.3 and 9.8%, and 1.4 and 4.3%, for the low and high levels of the standard curve, respectively.

### Statistical analysis

Data are expressed as mean ± SD. The variables among groups were compared using Student’s T-test, based on Shapiro-Wilk normality test. Differences were considered statistically significant at *P* < 0.05. All analyses were performed using the SigmaStat (v 11.00) package (SPSS, Inc., IL, USA).

## Results

### Body weight, ovarian size and ovarian histology

Body weights (BW) and ovarian weights (OW) of IUGR and *sham* animals are shown in Table 1.

**Table 1:** Body weight, ovarian weight and their ratio in IUGR and *sham* rats

	5 d*pp*			20 d*pp*			40 d*pp*		
	IUGR (*n* = 8)	*Sham* (*n* = 4)	*P*	IUGR (*n* = 6)	*Sham* *(n = 6)*	*P*	IUGR *(n = 6)*	*Sham* *(n = 6)*	*P*
BW (g)	10.6 ± 0.9	12.4 ± 0.5	**0.004**	55.8 ± 6.8	57.7 ± 6.3	0.662	152.1 ± 22.6	159.1 ± 15.4	0.628
OW × 10^^3^ (g)	2.5 ± 1.4	2.7 ± 0.6	0.933	19.2 ± 4.7	16.1 ± 7.6	0.394	46.0 ± 15.0	55.9 ± 8.81	0.234
OW:BW × 10^^3^ (g/g)	0.2 ± 0.1	0.2 ± 0.0	0.808	0.3 ± 0.1	0.3 ± 0.1	0.818	0.3 ± 0.0	0.3 ± 0.1	0.234

Values are expressed as means ± standard deviations. BW, body weight; d*pp*, days *post-partum*; IUGR, intrauterine growth restricted; OW, ovarian weight

Bold characters emphasize significant *P*-values

Mean BW in IUGR rats was still significantly reduced at 5 d*pp* compared to *sham* rats but caught up by 20 d*pp*. Ovarian weight (OW) and OW normalized per body weight (OW:BW) were not significantly different in the two groups (Table 1).

**Fig. 1:**
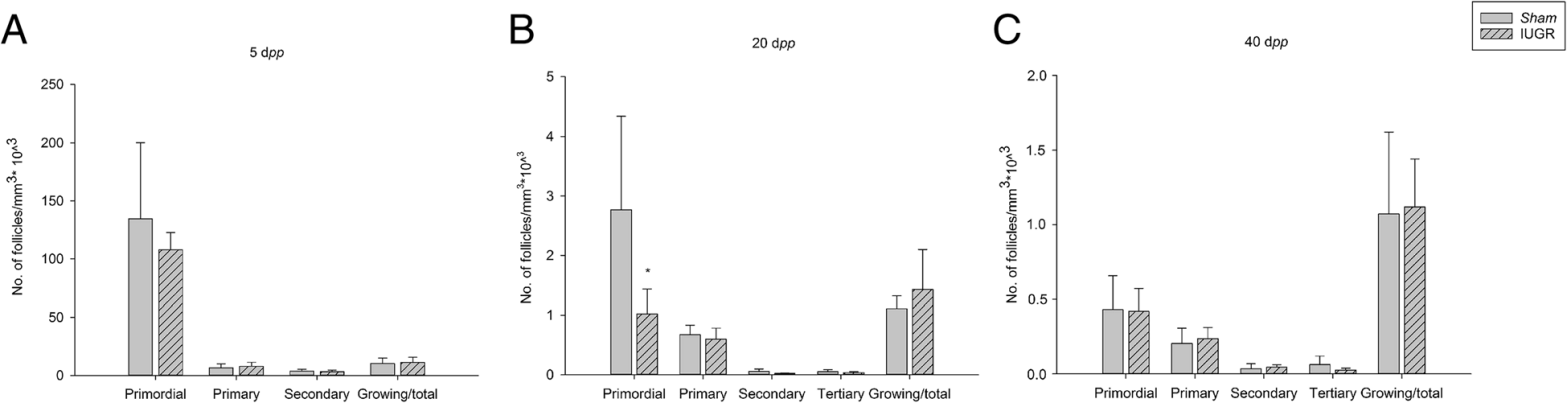
Densities of follicles in different developmental stages (primordial, primary, secondary and tertiary) and ratio of growing follicles / total follicles in intrauterine growth restricted (IUGR) and *sham* rats at **a** 5, **b** 20 and **c** 40 days *post-partum* (d*pp*). At 5 d*pp*, 4 animals were included in each group. At 20 and 40 d*pp* 6 animals were included in each group. Student T-test, * *P* < 0.05

In both groups, PF density declined from 5 to 40 dpp, in line with the expected age-related decrease in the number of PF from birth to puberty, that has been well-described in other mammalian species, i.e. humans and primates [32–34] (Additional file 1: Figure S1A). However, in IUGR rats at 5 d*pp* PF and total follicle showed a tendency to be lower compared to controls, although the comparison did not reach statistical significance. At 20 d*pp*, the number of PF and total follicles was significantly reduced in the ovaries of IUGR rats compared to *sham* (Fig. 1).

At 40 d*pp* no differences in the number of follicles were noted between the two groups of animals.

The ratio between growing follicles (primary, secondary and tertiary) and total follicles was calculated in order to evaluate a possible change in activation of PF and their subsequent transition into the pool of growing follicles. No difference was detected in the ratio between the groups at any age (Fig. 1).

Extrapolated ovarian volumes and CL number was also comparable between the groups (data not shown).

### Networks of genes affected by IUGR

We analyzed the relative expression of a panel of 90 genes that were selected because of their known functions in the gonads (Table 2).

**Table 2:** Abbreviations and names of the significantly dysregulated genes in *sham* versus IUGR rats from the TLDA analysis

Gene symbol	Name
Amh	Anti-Mullerian hormone
Angpt1	Angiopoietin 1
Casp9	Caspase 9
Cdkn1B	Cyclin-Dependent Kinase Inhibitor 1B (P21, Cip1)
Cs	Citrate Synthase
Fgf4	Fibroblast Growth Factor 4
Fgf5	Fibroblast Growth Factor 5
Gata6	GATA binding protein 6
Igf1r	Insulin-Like Growth Factor 1 Receptor
Igfbp3	Insulin-like growth factor binding protein 3
Inhbb	Inhibin subunit beta B
Insl3	Insulin-like 3
Ldhb	Lactate Dehydrogenase b
Ldhc	Lactate Dehydrogenase C
Mki67	Marker of proliferation Ki-67
Pcna	Proliferating cell nuclear antigen
Pdgfra	Platelet derived growth factor receptor alpha
Slc2a1	Solute Carrier Family 2 (Facilitated Glucose Transporter), Member 1
Tgfb1	Transforming growth factor, beta 1
Tgfb2	Transforming growth factor, beta 2
Thy1	Thy-1 Cell Surface Antigen
Tk1	Thymidine Kinase 1, Soluble
Top2a	DNA topoisomerase II alpha
Zbtb16	Zinc finger and BTB domain containing 16

*IUGR* intrauterine growth restricted, *TLDA* TaqMan low density array

In IUGR ovaries, 24 genes were differentially regulated as compared to controls (Table 3).

**Table 3:** Significant gene expression changes between *sham* and IUGR rats at 5, 20 and 40 d*pp*

Cluster	Gene	*Sham* vs IUGR		
		d*pp* 5	d*pp* 20	d*pp* 40
Proliferation	*Mki67*			1.0 ± 0.3 vs 0.5 ± 0.2
	*Pcna*			1.0 ± 0.1 vs 0.6 ± 0.2
	*Cdkn1b*			1.0 ± 0.3 vs 0.7 ± 0.1
	*Top2a*			1.0 ± 0.2 vs 0.4 ± 0.1
	*Tk1*			1.1 ± 0.6 vs 0.4 ± 0.2
Apoptosis	*Casp9*			1.0 ± 0.1 vs 0.8 ± 0.1
Energetics	*Cs*			1.0 ± 0.2 vs 0.8 ± 0.1
	*Ldhb*		1.0 ± 0.2 vs 1.3 ± 0.3	
	*Ldhc*	1.0 ± 0.3 vs 0.6 ± 0.1		
	*Slc2a1*			1.0 ± 0.3 vs 1.6 ± 0.5
Angiogenesis	*Tgfb1*	1.0 ± 0.1 vs 0.7 ± 0.1		
	*Tgfb2*		1.0 ± 0.3 vs 0.7 ± 0.3	
	*Angpt1*		1.0 ± 0.1 vs 0.8 ± 0.3	
	*Pdgfra*		1.0 ± 0.1 vs 1.4 ± 0.1	
Insulin/IGF pathways	*Igf1r*		1.0 ± 0.3 vs 0.8 ± 0.1	1.0 ± 0.2 vs 0.8 ± 0.1
	*Igfbp3*			1.0 ± 0.4 vs 3.0 ± 0.9
Germ cell markers	*Fgf4*	1.1 ± 0.4 vs 0.6 ± 0.2		
	*Fgf5*		1.5 ± 1.5 vs 0.2 ± 0.1	
	*Thy1*	1.0 ± 0.3 vs 1.4 ± 0.2		
	*Zbtb16*	1.1 ± 0.5 vs 0.5 ± 0.2		
GC markers	*Amh*		1.0 ± 0.1 vs 0.8 ± 0.1	
	*Inhbb*			1.0 ± 0.1 vs 0.5 ± 0.3
	*Gata6*			1.0 ± 0.2 vs 0.7 ± 0.1
TC markers	*Insl3*		1.1 ± 0.6 vs 3.0 ± 1.9	

Values are expressed as means ± standard deviations of gene expression fold changes. D*pp*, days *post-partum*; GC, granulosa cell; IGF, insulin growth factor; IUGR, intrauterine growth restricted; TC, theca cell. Student T-test, *P* < 0.05. For the list of gene names and abbreviation please refer to Table 2. At the age of 5 d*pp*, 6 IUGR versus 3 *sham* rats were included. At 20 and 40 d*pp*, 6 animals *per* group were included

**Fig. 2:**
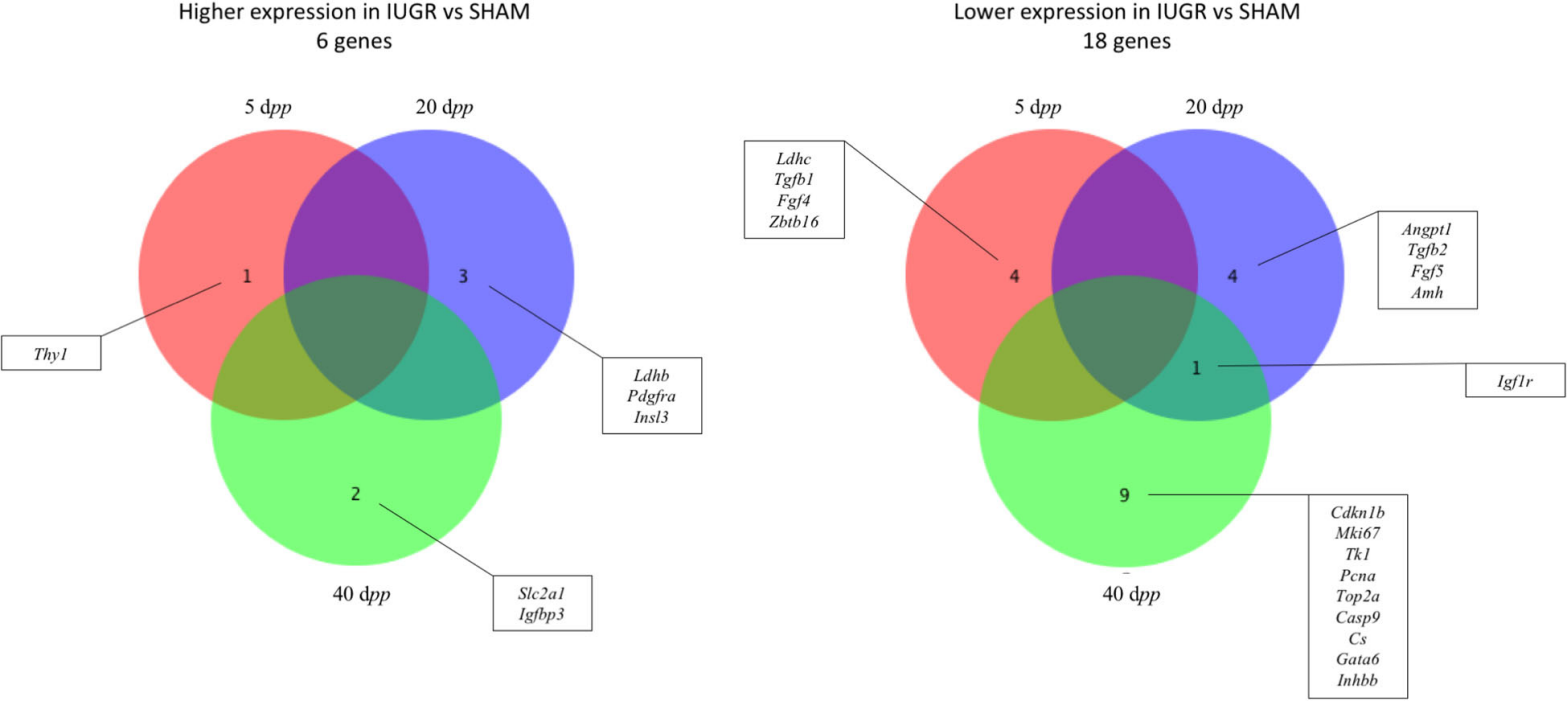
Venne diagrams representing genes with significantly higher and lower expression in intrauterine growth restricted (IUGR) vs *sham* rats at 5, 20 and 40 days *post-partum* (d*pp*) from TaqMan Low-Density Array analysis. Gene abbreviations and corresponding names are listed in Table 2

Among those, six genes showed a significantly higher expression and 18 genes showed a significantly lower expression in IUGR versus *sham* animals (Fig. 2).

Six genes involved in cell proliferation, survival and cycle regulation were significantly dysregulated in IUGR rats at 40 d*pp* compared to controls. In addition, five genes were modified concordantly towards a reduction in the proliferation rate of ovarian cells, namely Proliferation-related Ki67 antigen (*MKi67*), Proliferating cell nuclear antigen (*Pcna*), Cyclin-dependent kinase inhibitor 1B (*Cdkn1b*), DNA topoisomerase II (*Top2a*) and Thymidine kinase 1 (*Tk1*). The gene encoding for Caspase 9 (*Casp9*) was also downregulated at this age.

Three genes involved in ovarian angiogenesis were dysregulated in 20 d*pp* IUGR ovaries compared to *sham* ones, Angiopoietin 1 (*Angpt1*) and Transforming growth factor beta 2 (*Tgfb2*) expression was reduced, whereas Platelet derived growth factor receptor alpha polypeptide (*Pdgfra*) was upregulated.

Furthermore, IUGR animals showed a lower expression of Insulin-like growth factor (IGF) 1 receptor (*Igf1r*) both at 20 and 40 d*pp*, that together with the increased expression of IGF binding protein (*Igfbp3*) at 40 d*pp* pointed towards a downregulation of the IGF signaling within the ovary of IUGR rats.

The expression of four genes involved in the regulation of cellular metabolism was altered in IUGR animals at different time points, as compared to *sham*; in particular Lactate dehydrogenase-c (*Ldh-c*) and Citrate synthase (*Cs*) were downregulated at 5 d*pp* and 40 d*pp*, respectively. In IUGR rats at 20 d*pp Ldh-b* and *Ldh-c* were both upregulated and Solute carrier family 2 member 1 (*Slc2a1*) was upregulated at 40 d*pp*.

**Fig. 3:**
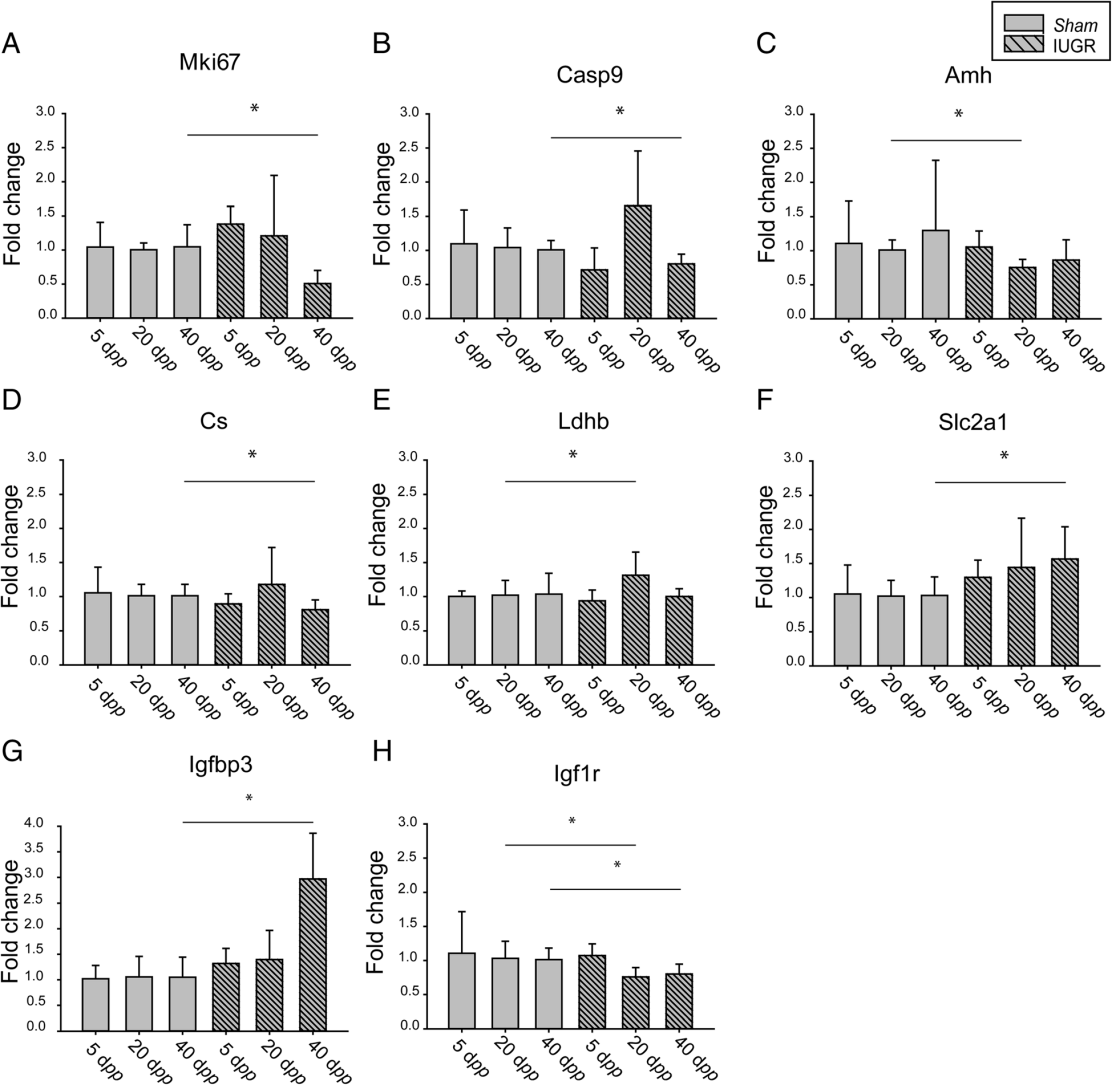
Relative gene expression of selected genes in intrauterine growth restricted (IUGR) and *sham* rats at 5, 20 and 40 days *post-partum* (d*pp*): **a** Mki67, marker of proliferation Ki-67, **b** Casp9, caspase 9, **c** Amh, anti-Mullerian hormone, **d** Cs, citrate synthase, **e** Ldhb, lactate dehydrogenase b, **f** Slc2a1, solute carrier family 2 (facilitated glucose transporter), member 1, **g** Igfbp3, insulin-like growth factor binding protein 3, **h** Igf1r, insulin-like growth factor 1 receptor. At 5 d*pp* 3 and 6 animals were included in the *sham* and IUGR group, respectively. At 20 and 40 d*pp* 6 animals were included in each group. Student T-test, * *P* < 0.05

Gene encoding for AMH (*Amh*) showed a reduced expression in IUGR ovaries at 20 d*pp* compared to controls. Inhibin B (*Inhbb*) expression was also lower at 40 d*pp* in IUGR rats versus *sham* ones. In Fig. 3 the expression of 8 representative genes from IUGR and sham ovaries at the three age points is reported.

### AMH assessment

Given the role of AMH as an indirect marker of ovarian reserve [35], we evaluated serum AMH levels in the two groups of animals. No significant difference was detected although a tendency to lower AMH concentrations in IUGR rats at all ages was observed (Additional file 1: Figure S1B).

In order to analyze whether a tendency to lower serum AMH and a lower expression of the gene encoding for AMH was related to a lower protein synthesis from the follicles, expression of ovarian AMH was evaluated by IHC; the analysis did not show any clear difference in the intensity of protein expression in granulosa cells between the groups (Additional file 1: Figure S1C).

## Discussion

Fetal life is the first fundamental window of susceptibility to pathogenic *noxae* for the individual. Events occurring during this period have been associated with gene reprogramming and modification of organ functions that can persist throughout the entire lifespan. Using the same animal model, we have previously demonstrated that in utero growth restriction altered size and morphology of the testis in neonatal rats and induced modifications of gene expression up to the peripubertal age [24]. Here, for the first time, we provide evidence of the effects of UPI during late gestation on the ovarian follicular pool and on the expression of genes involved in ovarian cellular processes.

In this study, we observed that the number of PF, constituting the ovarian reserve, was reduced at birth by fetal hypoxia and continued to diminish until the age of 20 d*pp*. In addition, we were able to demonstrate that UPI during late gestation induced alterations in the expression of multiple genes in the neonatal, prepubertal and peripubertal rat ovary.

A decrease in the number of follicles has been reported in rat models where IUGR was induced by different means, from maternal food restriction [22] to inflammation [36]. Our study is concordant with a previous study from Engelbregt and colleagues that observed a decrease in total, primordial and growing follicles in adult rats with IUGR induced by uterine artery ligation [25] and it is the first exploring changes in the ovary throughout postnatal development.

A reduction in the number of PF has also been detected in the ovaries of human fetuses who had restricted growth [37]. Poor growth in utero has been associated to an increased incidence of polycystic ovarian syndrome (PCOs) that may in turn cause reduced fertility [9, 38]. Despite some studies suggest that adverse events during fetal life can induce premature ovarian failure in women, data in this respect remain inconclusive [14]. In this frame, our study provides evidence of a qualitative and quantitative influence of fetal growth restriction on the ovarian follicle pool.

Despite the lower follicle number at prepubertal ages, we could not see any difference in follicle counts at the age of 40 d*pp*, indicating that long-term ovarian reserve was not affected and eventually that fertility was preserved after hypoxia occurring during late gestation. Recent studies in mice have raised the theory that two different classes of PF exist in the ovary: i) a “first wave” of PF, that is activated immediately after birth, persists in the mouse ovary up to ~ 3 months and contributes to the onset of puberty and to the early fertility; ii) an “adult wave” of PF, that is activated gradually after puberty and provides fertility until the end of reproductive life [39–41]. In this view, it is intriguing to speculate that late gestation hypoxia in our model might have affected the first wave of PF while leaving the adult pool unaffected. A lower number of PF from the first wave at 20 d*pp* could therefore explain the decreased number of PF observed at that age, which was no more evident at 40 d*pp* when PF from the non-affected adult wave become more prevalent.

The existence of two populations of follicles with different temporal distribution and susceptibility to *pathogenic noxae* would represent a fascinating biological mechanism. It would indeed imply that the developing ovary retain a compensatory capacity to ovarian damages which is lost as soon as the first wave of follicles is exhausted (i.e. after puberty). Presently there are no histological markers to distinguish the two populations of follicles.

The existence of ovarian stem cells (OSCs) has been debated in the last 15 years, since Tilly’s group claimed the detection of ovarian germ cell progenitors in the ovary [42–44]. Their findings have raised controversy and several studies have tried to address the issue with contradictory results [45]. Hence, an indisputable proof of the existence of these cells in the mammalian ovary and a protocol able to identify OSCs is still missing [45].

Nevertheless, the hypothesis that these cells reside in the ovary in a quiescent state and are activated in response to oocyte damage is still subjected to investigation and offer an intriguing alternative explanation to our findings.

Another interesting mechanism for the comparable PF density between groups at day 40 could be a lower rate of follicular attrition in IUGR rats between 20 and 40 d*pp* of age compared to *sham* ones. This phenomenon could be driven by the gene modifications observed in IUGR rats. Consistently, the expression of the gene encoding for CASPASE 9, a significant protein in the apoptotic cascade, was significantly downregulated at day 40 in IUGR rats compared to controls. In addition, previous studies in knock-out mice models have suggested that the Transforming growth factor beta 2 (TGFB2) has a role in the mediation of oocyte apoptosis during the embryonic stage. In our IUGR rats, expression of *Tgfb2* was reduced at day 20 allowing us to speculate of a possible implication of this factor in the control of apoptosis also during postnatal life.

Furthermore, increased activation and recruitment of PF into the growing pool has been proposed, in other pathogenic frames, as a mechanism that can eventually lead to ovarian reserve exhaustion [46]. An increased entry of PF into the pool of growing follicles could justify their reduction at day 20. However, we were not able to show any increase in the activation rate of PF, based on the ratio between growing follicles and total follicles. This was consistent with the expression of 5 different genes involved in cell proliferation (i.e. *Cdkn1b, Mki67, Tk1, Pcna, Top2a*) that was reduced in IUGR rat ovaries at 40 d*pp*. These changes could reflect a lower activation rate of PF, ultimately compensating the PF loss induced by the UPI. The same significance could be attributed to the modifications of genes belonging to the IGF system: the reduction of *Igf1r* at both day 20 and 40 and the parallel increase in *Igfbp3* expression at day 40 predicts a reduction in the activity of IGF-I, one of the most important growth factors, within the ovary.

Finally, some of the gene modifications occurring at 20 d*pp* and not observed at day 40, could reflect the shift from lactation to standard rat chow diet, as weaning happened coincidentally (21 d*pp*). Although most of the studies have focused on non-physiological dietary regimens (i.e. food restriction or high-fat diet) to test the effects on gene expression in different organs, normal weaning was shown to alter the mRNA levels of 18 genes in piglet small intestine [47].

Our study has a few potential limitations. First, pubertal onset and progression and sex steroid hormone profile were not evaluated in the experimental animals. Given the hypothesis that follicles from the first wave contribute to the onset of puberty and to the establishment of the hypothalamus-pituitary-ovarian axis, this information could have been supportive to the data. Second, we did not assess fertility in IUGR rats by verifying mating outcomes. Whether a normal number of follicles at peripubertal age reflects an intact fertility potential and above all is maintained throughout the reproductive lifespan in rats exposed to fetal hypoxia remains to be ascertained. Third, due to the limited amount of tissue available, it was not possible to verify gene expression data at the protein level, therefore results of our study have to be interpreted carefully, bearing in mind that post-transcriptional modifications could alter the functional outcome. The method we used to generate IUGR leads to a rapid disruption of ovarian perfusion by uterine artery ligation in late gestation, which is different from the gradual onset of hypoxia that may occur more slowly during the human pregnancy. Furthermore, we investigated the effects of a late-onset placental insufficiency, whereas human fetal growth restriction is often the consequence of a disrupted placental development with earlier and sustained onset. Therefore, despite these data appear reassuring, more studies are required to investigate if the full reproductive lifespan is affected in the human pathogenic setting. Finally, it has to be pointed out that all animals showed early postnatal catch-up growth, normalizing their body size soon after birth. Therefore, the fate of ovaries in animals that remain permanently small (as occurs in almost 10% of children born SGA) remains unknown. Inter-species differences between rats and humans need also to be taken into account when translating these results to human pathology.

## Conclusions

In conclusion, we demonstrated that IUGR induced by late gestation hypoxia affects the number of follicles in juvenile rats, in particular targeting primordial follicles. The recovery in follicle number observed at peripubertal ages could be regulated by different mechanisms, putatively involving gene reprogramming, although data in this respect remain preliminary. Despite these results appear reassuring, at least when considering late-onset placental insufficiency, long-term longitudinal studies are required to investigate whether the full reproductive lifespan is affected.

## Additional file

Additional file 1: **Figure S1.** A) Density of primordial follicles in intrauterine growth restricted (IUGR) and *sham* rats at 5, 20 and 40 days *post-partum* (d*pp*). Student T-test, * *P* < 0.05. B) Serum levels of anti-Müllerian hormone (AMH) (ng/ml) in IUGR and *sham* rats at 5, 20 and 40 d*pp*. At 5 d*pp* 4 and 7 animals were included in the *sham* and IUGR group, respectively. At 20 d*pp* 6 animals were included in each group. At 40 d*pp* 2 and 6 animals were included in the *sham* and IUGR group, respectively. C) Immunohistochemical images of AMH in two representative sections for each age (5, 20 and 40 d*pp*) in ovaries from IUGR and *sham* rats. Scale bars correspond to 100 μm for 5 d*pp* animals and to 500 μm for 20 and 40 d*pp* animals (DOCX 334 kb)

## Abbreviations

AGA: Appropriate for gestational age
Am: Area of the middle section
AMH: Anti-Müllerian hormone
Angpt1: Angiopoietin 1
BW: Birth weight
Casp9: Caspase 9
Cdkn1b: Cyclin-dependent kinase inhibitor 1B
CL: *Corpus luteum*
DOHaD: Developmental origins of health and disease’ hypothesis
D*pp*: Days *post-partum*
GC: Granulosa cells
IGF: Insulin-like growth factor
Igf1r: IGF receptor 1
Igfbp3: IGF binding protein 3
IHC: Immunohistochemistry
Inhbb: Inhibin B
IUGR: Intrauterine growth restriction
Ldh-b: Lactate dehydrogenase-b
Ldh-c: Lactate dehydrogenase-c
MKi67: Proliferation-related Ki67 antigen
OSCs: Ovarian stem cells
OW: Ovarian weight
Pcna: Proliferating cell nuclear antigen
PCOs: Polycystic ovarian syndrome
Pdgfra: Platelet derived growth factor receptor alpha polypeptide
PF: Primordial follicles
SDS: Standard deviations
SGA: Small for gestational age
Slc2a1: Solute carrier family 2 member 1
TBS: Tris-buffered saline
Tgfb2: Transforming growth factor beta 2
Tk1: Thymidine kinase 1
TLDA: TaqMan low density array
Top2a: DNA topoisomerase II
UPI: Uteroplacental insufficiency
V: Volume
