# Supplementary material for: Impact of uteroplacental insufficiency on ovarian follicular pool in the rat
Source: Reprod Biol Endocrinol. 2019 Jan 10;17:10. doi: 10.1186/s12958-019-0453-3 (PMC6329190; doi:10.1186/s12958-019-0453-3)
Supplement: Supplementary file 1 — Figure S1. A) Density of primordial follicles in intrauterine growth restricted (IUGR) and sham rats at 5, 20 and 40 days post-partum (dpp). Student T-test, * P < 0.05. B) Serum levels of anti-Müllerian hormone (AMH) (ng/ml) in IUGR and sham rats at 5, 20 and 40 dpp. At 5 dpp 4 and 7 animals were included in the sham and IUGR group, respectively. At 20 dpp 6 animals were included in each group. At 40 dpp 2 and 6 animals were included in the sham and IUGR group, respectively. C) Immunohistochemical images of AMH in two representative sections for each age (5, 20 and 40 dpp) in ovaries from IUGR and sham rats. Scale bars correspond to 100 μm for 5 dpp animals and to 500 μm for 20 and 40 dpp animals (DOCX 334 kb) [file 12958_2019_453_MOESM1_ESM.docx]

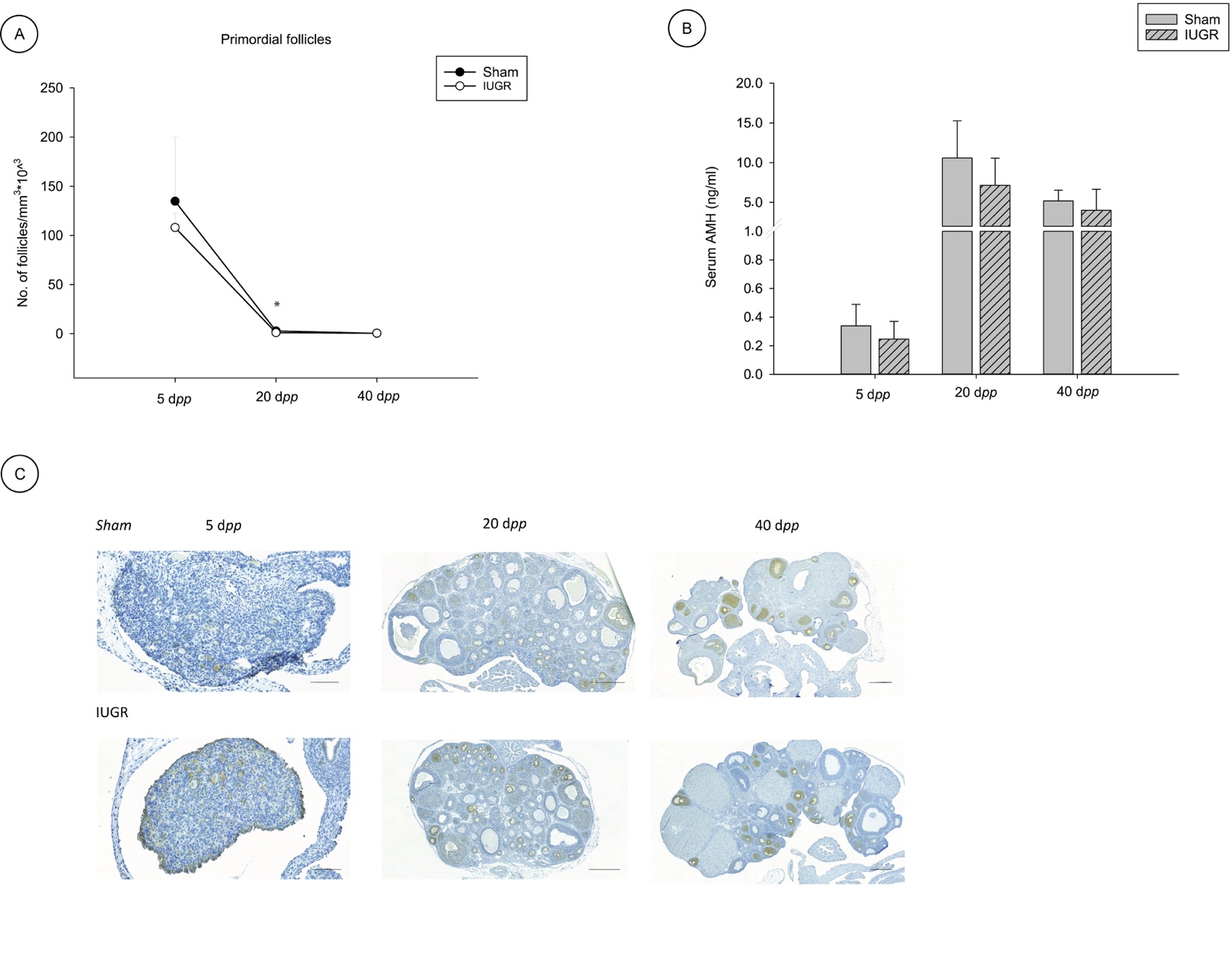


**Figure S1.** A) Density of primordial follicles in intrauterine growth restricted (IUGR) and *sham* rats at 5, 20 and 40 days *post-partum* (d*pp*). Student T-test, * P < 0.05. B) Serum levels of anti-Müllerian hormone (AMH) (ng/ml) in IUGR and *sham* rats at 5, 20 and 40 d*pp*. At 5 d*pp* 4 and 7 animals were included in the *sham* and IUGR group, respectively. At 20 d*pp* 6 animals were included in each group. At 40 d*pp* 2 and 6 animals were included in the *sham* and IUGR group, respectively. C) Immunohistochemical images of AMH in two representative sections for each age (5, 20 and 40 d*pp*) in ovaries from IUGR and *sham* rats. Scale bars correspond to 100 µm for 5 d*pp* animals and to 500 µm for 20 and 40 d*pp* animals.
